# Supplementary material for: The efficiency and safety of alendronate versus teriparatide for treatment glucocorticoid-induced osteoporosis: A meta-analysis and systematic review of randomized controlled trials
Source: PLoS One. 2022 May 31;17(5):e0267706. doi: 10.1371/journal.pone.0267706 (PMC9154179; doi:10.1371/journal.pone.0267706)
Supplement: S2 File — (ZIP) [file pone.0267706.s002.zip › search strategies.docx]

**Search strategy in PubMed**

#5 Select 1 document(s)

#4 Search ((alendronate) AND Teriparatide) AND ((((((((((glucocorticoid) OR corticosteroid) OR methylprednisolone) OR prednisone) OR prednisolone) OR hydrocortisone) OR triamcinolone) OR dexamethasone) OR corticoid) AND Osteoporosis) Filters: Randomized Controlled Trial; Humans Sort by: [pubsolr12]

#3 Search (((((((((glucocorticoid) OR corticosteroid) OR methylprednisolone) OR prednisone) OR prednisolone) OR hydrocortisone) OR triamcinolone) OR dexamethasone) OR corticoid) AND Osteoporosis Filters: Randomized Controlled Trial; Humans Sort by: [pubsolr12]

#2 Search (((((((((glucocorticoid) OR corticosteroid) OR methylprednisolone) OR prednisone) OR prednisolone) OR hydrocortisone) OR triamcinolone) OR dexamethasone) OR corticoid) AND Osteoporosis Filters: Humans Sort by: [pubsolr12]

#1 Search (((((((((glucocorticoid) OR corticosteroid) OR methylprednisolone) OR prednisone) OR prednisolone) OR hydrocortisone) OR triamcinolone) OR dexamethasone) OR corticoid) AND Osteoporosis Sort by: [pubsolr12]

**Search strategy in Embase**

#10. #9 AND 'randomized controlled trial'/de

#9. #4 AND #8

#8. teriparatide AND alendronate

#7. #5 AND 'randomized controlled trial topic'/de

#6. #5 AND 'randomized controlled trial topic'/de

#5. (glucocorticoid OR corticoid OR corticosteroid OR

methylprednisolone OR prednisone OR prednisolone

OR hydrocortisone OR triamcinolone OR

dexamethasone) AND osteoporosis AND alendronate

AND teriparatide

#4. (glucocorticoid OR corticoid OR corticosteroid OR

methylprednisolone OR prednisone OR prednisolone

OR hydrocortisone OR triamcinolone OR

dexamethasone) AND osteoporosis AND randomized

AND controlled AND trial

#3. #1

#2. #1 AND 'randomized controlled trial'/de

#1. 'glucocorticoid induced' AND osteoporosis AND

teriparatide AND alendronate AND randomized AND

controlled AND trial

**Search strategy in The Cochrane Library**

17 Trials matching glucocorticoid or corticosteroid or methylprednisolone or prednisone or prednisolone or hydrocortisone or triamcinolone or dexamethasone in All Text AND teriparatide in All Text AND alendronate in All Text AND randomized controlled trial in All Text AND Osteoporosis in All Text - (Word variations have been searched).

**Search strategy in Web of Science**

((((((((((TS=(glucocorticoid)) OR TS=(corticosteroid)) OR TS=(methylprednisolone)) OR TS=(prednisone)) OR TS=(prednisolone)) OR TS=(hydrocortisone)) OR TS=(triamcinolone)) OR TS=(dexamethasone)) AND TS=( teriparatide)) AND TS=(alendronate )) AND TS=(Osteoporosis)

**Search strategy in Google Scholar**

glucocorticoid or corticosteroid or methylprednisolone or prednisone or prednisolone or hydrocortisone or triamcinolone or dexamethasone and Osteoporosis and teriparatide and alendronate.
