# Supplementary material for: The efficiency and safety of alendronate versus teriparatide for treatment glucocorticoid-induced osteoporosis: A meta-analysis and systematic review of randomized controlled trials
Source: PLoS One. 2022 May 31;17(5):e0267706. doi: 10.1371/journal.pone.0267706 (PMC9154179; doi:10.1371/journal.pone.0267706)
Supplement: Attachment — Submitted filename: Response to Reviewers.docx [file pone.0267706.s003.docx]

Dear Editor and Reviewers:

Thank you for your letter of Manuscript ID PONE-D-22-00100 entitled " The efficiency and safety of alendronate versus teriparatide for treatment glucocorticoid-induced osteoporosis: A

meta-analysis and systematic review of randomized controlled trials" and for the reviewer’s comments concerning our manuscript.

We have carefully studied your amendments. After careful study and discussion by all our authors, and given the amendments put forward by you, we reply as follows:

**Journal Requirements:**

Comment 1: Please ensure that your manuscript meets PLOS ONE's style requirements, including those for file naming.

Reply 1: We have modified the format of the manuscript to meet the style requirements of PLOS ONE.

Comment 2: Financial disclosure.

Reply 2: Regarding the sources of funding, our answer is “The authors received no specific funding for this”

Comment 3: Conflict of interest

Reply 3: On conflicts of interest, our answer is “The authors have declared that no competing interests exist.”

Comment 4: Data Availability statement

Reply 4: We have uploaded the study’s minimal underlying data set for the study as a supporting information file.

**Reviewers' issues:**

**Reviewer #1**

Comment 1: Grey literature

Reply 1: We used several popular grey literature retrieval websites and found that there were no new articles that met the inclusion criteria. The websites include <http://www.opendoar.org/>、<https://www.openaire.eu/>、<http://www.base-search.net/about/en/>、

<https://zetoc.jisc.ac.uk/> 、<https://ethos.bl.uk/Home.do、http://www.dart-europe.eu/basic-search.php>, and<http://OpenDissertations.org>.

Comment 2: The protocol registration was absent.

**Reply 2**: We have already conducted the protocol registration, but the last submission did not include this part. The specific information of the protocol registration is as follows: Our registration number is INPLASY202090025. Our DOI number is 10.37766/inplasy2020.9.0025. The web address is https://inplasy.com/

Comment 3: independent investigators

Reply 3: In the “Methods section”, we have identified the primary investigators for data extraction and quality assessment for each included study.

Comment 4: search strategies

Reply 4: “P” in this study refers to the patients with osteoporosis caused by glucocorticoid administration, so the “Mesh Term” of our search term are “glucocorticoid” and “osteoporosis”, “Entry Terms” are “glucocorticoids, corticoid, corticoids, corticosteroids, corticosteroid, methylprednisolone, prednisone, prednisolone, hydrocortisone, triamcinolone, dexamethasone”.

“I” refer to the treatment of glucocorticoid-induced osteoporosis with two different drugs; This study is specific to two drugs, so the search terms are "alendronate" and "teriparatide."

Comment 5: Exact date and duration of the literature search in both abstract and main manuscript

Reply 5: The time for literature retrieval in this study was indeed outdated, so all the authors of this article conducted literature retrieval again. Unfortunately, the literature that met the inclusion criteria did not increase, that is, no new relevant study was published since March 2021.

Comment 6: How authors dealt with missing data.

Reply 6: Some of the data were reasonably deduced by us, while others were contacted by the authors of the original study in various ways, but the authors did not reply.

Comment 7: It is recommended to add a funnel plot.

Reply 7: We used Stata 16.0 to draw the funnel plot.

Comment 8: Linguistic problems

Reply 8: Before submitting the manuscript this time, we also invited a professional polishing company to make linguistic modifications to the manuscript.

**Reviewer #2**

Comment 1: Effect of oral glucocorticoid on the incidence of fracture.

Reply 1: We looked carefully at the original literature, and we did have a problem with what we described in the manuscript, the results of the original study showed that hip fracture rates were similar in low-dose corticosteroid users and controls, but there was a 77% increase in fracture rates at daily oral corticosteroid doses of 2.5-7.5mg and a 127% increase at doses of 7.5 mg or higher. So, we've made changes to this part of the manuscript.

Comment 2: Inaccurate expression

Reply 2: After discussion by our authors, we feel that the revised scheme you put forward is more accurate and concise in expressing the views we want to clarify.

Comment 3: Repetitive statements/conclusion

Reply 3: What we have written in this part is indeed repeated, and the repeated expressions have been removed in the corresponding part of the manuscript.

Comment 4: Repetitive statements

Reply 4: This part of our manuscript does contain repetitions, and the repetitions have been removed.

Comment 5: the mechanism of GIOP and pathogenesis is discussed in great detail but it does not relate to this paper.

Reply 5: The manuscript does contain a large section on the pathogenesis of osteoporosis, and we have already condensed the relevant content.

Comment 6: Content expression is not standard enough.

Reply 6: We have made changes to the relevant parts of the manuscript.

Comment 7: Content expression is not standard enough.

Reply 7: We have deleted “kind of”.

Comment 8: Repetitive statements

Reply 8: We have removed the duplicate parts based on your suggestion.

Comment 9: Discuss the shortcomings in the current literature.

Reply 9: We have revised the discussion section of the manuscript.

Comment 10: The discussion part of the manuscript is not enough.

Reply 10: We have revised the discussion section of the manuscript.

Comment 11: Repetitive statements

Reply 11: We have removed the duplicate parts based on your suggestion.

Comment 12: The discussion should be well structured.

Reply 12: We rearranged the discussion.

Comment 13: inclusion and exclusion criteria

Reply 13: We have carefully read all five studies included in this article, and the Materials and Methods sections of all studies have clarified that the bone mineral density (BMD) T score of the lumbar spine or femoral neck of the patient was ≤-2 or <-1 plus at least one fragility fracture.

Comment 14: Repetitive statements

Reply 14: We removed duplicates from the results section of the manuscript.
