# Supplementary material for: The efficiency and safety of alendronate versus teriparatide for treatment glucocorticoid-induced osteoporosis: A meta-analysis and systematic review of randomized controlled trials
Source: PLoS One. 2022 May 31;17(5):e0267706. doi: 10.1371/journal.pone.0267706 (PMC9154179; doi:10.1371/journal.pone.0267706)
Supplement: Attachment — Submitted filename: Response to Reviewers - 2.docx [file pone.0267706.s004.docx]

Dear Editor and Reviewers:

Thank you for your letter of Manuscript ID PONE-D-22-00100R1 entitled " The efficiency and safety of alendronate versus teriparatide for treatment glucocorticoid-induced osteoporosis: A meta-analysis and systematic review of randomized controlled trials" and for the reviewer’s comments concerning our manuscript.

We have carefully studied your amendments. After careful study and discussion by all our authors, and given the amendments put forward by you, we reply as follows:

**Academic editor:**

Our expert reviewer(s) have recommended some minor revisions to your manuscript. Please also include timeline of the literature search in the method section of the abstract. Therefore, I invite you to respond to the reviewer(s)' comments as below and revise your manuscript.

**Reply**: We have added timeline for literature search to the modified manuscript abstract section.

**Reviewers' issues:**

**Reviewer #1**

Comment: Did the search strategy the same in all databases? Search terms in PubMed and Embase are different. The authors should attach syntax used in each database in supplementary.

Reply: The search strategies of each database do vary, and we have uploaded the detailed search strategies of these databases as supplementary.
